# Are Prophylactic Systemic Antibiotics Required in Patients with Cataract Surgery at Local Anesthesia?

**DOI:** 10.3390/ijerph192315796

**Published:** 2022-11-27

**Authors:** Toshihiko Matsuo, Masahiro Iguchi, Noriyasu Morisato, Tatsuya Murasako, Hideharu Hagiya

**Affiliations:** 1 Graduate School of Interdisciplinary Science and Engineering in Health Systems, Okayama University, Okayama 700-8558, Japan; 2 Department of Ophthalmology, Okayama University Hospital, Okayama 700-8558, Japan; 3 Eye Clinic, Ochiai Hospital, Maniwa 719-3197, Japan; 4 Department of Pharmacy, Ochiai Hospital, Maniwa 719-3197, Japan; 5 Clinical Laboratories, Ochiai Hospital, Maniwa 719-3197, Japan; 6 Department of General Medicine, Okayama University Hospital, Okayama 700-8558, Japan; 7 Graduate School of Medicine, Dentistry, and Pharmaceutical Sciences, Okayama University, Okayama 700-8558, Japan

**Keywords:** cataract surgery, global action plan, antimicrobial resistance, appropriate use, antibiotics, oral, intravenous, topical, povidone iodine, conjunctival sac culture

## Abstract

The reduced use of antimicrobial drugs has been recommended worldwide, according to the global action for antimicrobial resistance published in 2015 by the World Health Organization. In this study, we retrospectively reviewed the incidence of surgical site infection in consecutive patients with cataract surgeries at a single hospital in the 6-year process when prophylactic systemic antibiotics were reduced in a step-by-step manner. The entire study period from 2016 to 2022 was divided into five stages, based on the use of systemic antibiotics. In stage 1 with 649 cataract surgeries, an intravenous drip infusion of cefazolin 1 g was given at surgery, followed by oral cefdinir 100 mg in the evening on surgery day and three times for two postoperative days. In stage 2 with 541 cataract surgeries, oral cefdinir 100 mg was given in the late morning before surgery, in the evening, and three times (300 mg in total) for two postoperative days. In stage 3 with 103 cataract surgeries, oral levofloxacin 500 mg was given in the late morning before surgery and once in the morning for two postoperative days. In stage 4 with 545 cataract surgeries, oral levofloxacin 500 mg was given only in the late morning before surgery. In stage 5 with 311 cataract surgeries, no systemic antibiotics were given. As common procedures in all stages, moxifloxacin eye drops were given four times daily as topical antibiotics in the 3 days before surgery and about 2 weeks after surgery. At surgery, the ocular surface was frequently irrigated with saline-diluted povidone iodine at 0.5% working concentration. No postoperative infection was recorded in any stage. This study showed neither harm nor risk in reduced use and, consequently, no use of prophylactic systemic antibiotics in cataract surgery as far as local precautionary measures were secured.

## 1. Introduction

A global action plan for antimicrobial resistance was published in 2015 by the World Health Organization [1]. Subsequently, the guideline for the appropriate and optimized use of antimicrobial drugs in Japan was established by the Government of Japan [2]. Cataract surgery reached over 900,000 as the annual number of surgeries in the fiscal year 2020 from April 2020 to March 2021 and occupied the top position in the ranking list of surgeries performed in Japan [3]. A rare but devastating complication in cataract surgery is surgical site infection, which leads to exogenous bacterial endophthalmitis [4]. Since the eye globe is a closed space, bacterial endophthalmitis runs a rapid course to involve the retina and results in vision loss. Lawsuits have been filed against ophthalmologists in a hospital or an eye clinic to pursue compensation for patients’ vision that is damaged as a result of bacterial endophthalmitis after cataract surgeries [5,6]. According to the judicial precedents, which recommended prophylactic use of systemic antibiotics in cataract surgery, intravenous and oral antibiotics have been used as a routine for a long time in Japan [7].

Clinical evidence for the systemic administration of prophylactic antibiotics in cataract surgery has not been accumulated to date [7]. No worldwide consensus has been established regarding endophthalmitis prophylaxis in cataract surgery [4], and prophylactic systemic antibiotics are not given in cataract surgery in most countries, except for Japan [8]. To reduce the use of antibiotics according to the action plan for antimicrobial resistance [2], we discontinued the use of intravenous and oral antibiotics as prophylactic administration for cataract surgery in a step-by-step manner in the preceding years. In this study, we retrospectively reviewed consecutive patients with cataract surgery performed by a single surgeon at a single hospital to show evidence of neither harm nor risk in reduced use and, consequently, no use of prophylactic antibiotics in cataract surgery.

## 2. Materials and Methods

### 2.1. Study Design

This retrospective study involved 2149 consecutive cataract surgeries, either as day surgery or overnight stay surgery, in 1381 patients, performed by a single surgeon (T.M.) from April 2016 to October 2022 at Ochiai Hospital, a local hospital with 135 beds in Maniwa City with a population of about 43,000 in 2022, located in the northern part of Okayama Prefecture, Japan. Day surgery or overnight stay surgery was chosen by patients solely based on their wishes. The study was approved as a retrospective study by the Ethics Committee in Okayama University Graduate School of Medicine, Dentistry, and Pharmaceutical Sciences and Okayama University Hospital (Identifier, 2005-002) and by the Ethics Committee in Ochiai Hospital. Medical records were reviewed to obtain the age, gender, results of preoperative conjunctival sac culture, and systemic use of antibiotics. 

### 2.2. Preoperative Conjunctival Sac Culture

As a routine procedure, conjunctival sac culture in each eye with scheduled cataract surgery was submitted to clinical laboratories, usually one month before the scheduled date of surgery. Antimicrobial susceptibility testings for antibiotics were performed in cases of positive bacterial culture. Topical 0.5% moxifloxacin was given 4 times daily, 3 days before surgery, in patients with negative cultures, and also in patients with positive cultures, with the detected bacteria being susceptible to moxifloxacin. When the isolated organisms, for instance, Corynebacterium species, were resistant to moxifloxacin while susceptible to cephems, 0.5% cefmenoxime eye drops 4 times daily were prescribed for 3 days before surgery. In cases where methicillin-resistant staphylococci (*Staphylococcus aureus* and coagulase-negative staphylococci such as *Staphylococcus epidermidis*), which were resistant to both moxifloxacin and cephems, were detected, 0.5% arbekacin eye drops 4 times daily (prepared by diluting with saline 100 mg/2 mL arbekacin sulfate for injection) was given for a week and the second culture was repeated to prove negative.

### 2.3. Surgical Procedure

All 2149 cataract surgeries were carried out through the upper corneal incision with 2.4 mm width in the standard procedure of phacoemulsification and aspiration with intraocular lens implantation, except for extracapsular cataract extraction with intraocular lens implantation in 21 eyes and intracapsular cataract extraction with intraocular lens-suturing in 6 eyes. Combined resection of pterygium was performed in 14 eyes with cataract surgeries and combined trabeculotomy using an ab interno approach was performed in 8 eyes with cataract surgeries. Intravitreous injection of aflibercept or ranibizumab was performed at the end of cataract surgery in 5 eyes. No surgical complication was recorded. 

After instillation of topical 4% lidocaine, eyelid skin was disinfected with 10% povidone iodine, and the ocular surface was disinfected with 16-times saline-diluted 10% povidone iodine at the working concentration of 0.625%. The eye was draped with a surgical sheet, and eyelashes on the upper and lower eyelids were covered with a sheet of transparent film (3M Tegaderm) by the placement of an eyelid speculum. During the surgery, the ocular surface was frequently irrigated with 20-times saline-diluted 10% povidone iodine at the working concentration of 0.5%. In patients with iodine allergy, 0.1% chlorhexidine was used to disinfect the skin and saline-diluted chlorhexidine at the working concentration of 0.02% was used to wash the ocular surface before and during the surgery. After the surgery, 0.5% levofloxacin was applied topically to the ocular surface and then the 0.1% betamethasone ointment and 0.3% ofloxacin ointment were instilled. Gauze eyepatch was worn overnight and removed the next morning. In patients with poor vision or no vision in the unoperated-on fellow eye, protective transparent plastic eyewear was used in place of gauze eyepatch after the instillation of 0.5% levofloxacin only. The next day after surgery, all patients started to use a new bottle (5 mL) of 0.5% moxifloxacin 4 times daily, usually for two weeks, in combination with 0.1% bromfenac twice daily for a month. Topical 0.1% betamethasone was added 4 times daily in case of inflammation-prone conditions such as floppy iris syndrome, exfoliation syndrome, retinitis pigmentosa, diabetes mellitus, and the history of uveitis or iritis.

All the surgeries were performed at local anesthesia in the afternoon, and the patients skipped lunch. After the surgery, there were no limits on daily activity and patients took a shower below the neck if they wished. They were instructed not to wash their face and head on the day of surgery. They took a bath and washed their hair with shampoo from next day after the surgery. During the bath, they were instructed to wash their face with a warm shower of running tap water a week after the surgery. They were also instructed to wear protective glasses or their own glasses and not to touch the eye with their fingers in the daytime.

All the patients were followed-up at routine visits on the next day, 5 days, a week and two weeks after surgery to check for the development of complications such as iritis and a transient rise in the intraocular pressure.

## 3. Results

### 3.1. The Use of Systemic Antibiotics in Five Stages

The entire study period was divided into five stages, based on the use of systemic antibiotics (Table 1). In Stage 1, from April 2016 to 18 January 2018, intravenous drip infusion of cefazolin 1 g was given at the time of surgery, followed by oral cefdinir 100 mg in the evening on the day of surgery and three times for two postoperative days. In Stage 2 from 26 January 2018 to August 2019, 100 mg of oral cefdinir was given before the surgery in the late morning and also in the evening on the day of surgery, and three times (300 mg in total) for two postoperative days. Based on the estimated glomerular filtration rate (eGFR) in each patient, the daily dose of cefdinir was reduced to 200 mg and 100 mg, respectively, in the range of 59–30 mL/min/1.73 m^2^ and less than 30 mL/min/1.73 m^2^. In patients undergoing hemodialysis, the daily dose of cefdinir was set at 100 mg. In case of allergic history to cephems, 500 mg oral levofloxacin was given before surgery in the late morning on the day of surgery and once in the morning for two postoperative days as a common procedure in Stage 1 and 2. 

In Stage 3 from September 2019 to December 2019, 500 mg oral levofloxacin was given before the surgery in the late morning on the day of surgery and also once in the morning for two postoperative days. In patients with eGFR less than 30 mL/min/1.73 m^2^, 500 mg oral levofloxacin was given only once before the surgery in the late morning. In Stage 4, from January 2020 to October 2021, 500 mg oral levofloxacin was given before the surgery in the late morning only. In Stage 5 from November 2021 to October 2022, no systemic antibiotics were given.

### 3.2. Preoperative Conjunctival Sac Culture

Table 2 summarizes the results of conjunctival sac culture in each stage. The lists of bacterial strains were not very different among the five stages. In an overall trend, the percentage of positive bacterial cultures in the conjunctival sac remained flat from the earlier stages to the recent stage. The percentage of methicillin-resistant staphylococci in positive cultures also tended to be stable from the earlier stages to the recent stage.

### 3.3. Incidence of Postoperative Infection

The number of cataract surgeries performed in each stage is given in Table 1. No entire postoperative infection was recorded in any stage.

## 4. Discussion

Antibiotic prophylaxis has been performed in all disciplines of surgery for decades by the intravenous drip infusion of antibiotics to achieve the maximum concentration at the start of the surgery as a gold standard [9,10]. In the field of ophthalmic surgery, which usually uses local anesthesia, intravenous drip infusion of first-generation cephems such as cefazolin sodium 1 g has been the long-term standard [7]. In cases where patients have a history of allergic reactions to cephems, fluoroquinolones such as levofloxacin were alternatively administered as prophylaxis. Even with careful history-taking, we found that patients with cataract surgery occasionally developed skin rashes or hotness as probable allergic reactions to cephems. In this background, we discontinued the administration of intravenous cephems on the day of surgery and started to give oral cephems before surgery to achieve the maximum concentration at the very beginning of surgery, in addition to the same oral cephems for two days after surgery (Stage 1 to Stage 2). 

The use of oral cephems for 3 days, including the surgery day, was replaced by oral levofloxacin once in the morning from Stage 2 to Stage 3, considering the low (approximately 20%) oral availability of cefdinir due to its poor solubility in water, depending on pH [11,12]. We found that some patients complained of bowel troubles such as abdominal pain and diarrhea in the days after surgery, which were considered adverse events of oral antibiotics. We determined to provide a single dose of oral levofloxacin before surgery on the day of surgery (from Stage 3 to Stage 4). In Stages 1, 2, and 3, the clinical path for cataract surgery became complicated due to measures to stop intravenous cephems and switch from cephems to fluoroquinolones in case of allergy to cephems, and to adjust the dose of antibiotics according to the renal function in each patient.

In parallel with the systemic administration of prophylactic antibiotics in Stages 1, 2, 3, and 4, topical antibiotics for 3 days preceding the date of surgery continued as the gold standard for intraocular surgeries such as cataract, glaucoma, and vitreous surgery [7]. Prophylactic topical antibiotics are usually continued for 2 weeks after surgery. We chose moxifloxacin eye drops, since this drug has been approved for prophylactic use before intraocular surgeries in Japan. 

The most important aspect to prevent infection in intraocular surgeries is the disinfection of the ocular surface. In our hospital, saline-diluted povidone iodine at the working concentration of 0.5% (20-times dilution) or 0.625% (16-times dilution) was used to disinfect the ocular surface before the start of surgery following the eyelid skin disinfection with 10% povidone iodine. Around the year 2010 in Japan, repeat irrigation of the ocular surface in the surgery with saline-diluted povidone iodine at the working concentration of 0.5% (20-times dilution) or 0.25% (40-times dilution) become the standard in intraocular surgeries [13,14]. At present, saline-diluted povidone iodine at the working concentration of 0.25% (40-time dilution) is recommended as the standard to disinfect the ocular surface before the surgery and irrigate the ocular surface during surgery [13,14]. Under the circumstances, we decided to stop using prophylactic oral levofloxacin on the day of surgery (Stage 4 to Stage 5). Of course, topical moxifloxacin eye drops were continued before and after cataract surgery.

Conjunctival sac culture was performed before intraocular surgeries, especially to screen for methicillin-resistant staphylococci. Moxifloxacin eye drops are prescribed as a routine before the cataract surgery at this hospital. Older patients who experienced preceding hospitalization for other diseases are sometimes colonized with methicillin-resistant staphylococci as inhabitants in the conjunctival sac [15]. To avoid post-surgery infection with these organisms, we used arbekacin eye drops as prophylaxis. This procedure also intended to avoid the patients from bringing methicillin-resistant staphylococci inside the hospital in cases of overnight stay. We prefer arbekacin eye drops [16] to vancomycin eye drops [17] since the vancomycin eye drop solution has acidic pH, leading to irritation on the ocular surface. The stable trend of methicillin-resistant staphylococci in the conjunctival sac in this study is in marked contrast with the decreasing trend in the other hospital in a different area of Japan, which we reported previously [15].

In the present series of patients, cataract surgeries were performed through a 2.4 mm-wide upper corneal incision in the standard procedure of phacoemulsification and aspiration with intraocular lens implantation. On rare occasions, extracapsular or intracapsular cataract extraction with a 9-to-11 mm-wide sclerocorneal incision was chosen as a safe surgical procedure in the cases of weak lens capsule and brown hard lens nucleus in people over 80 years old or in the cases of weak ciliary zonules with lens subluxation, manifesting as a shallow anterior chamber. Intraocular lens-suturing was performed in intracapsular cataract extraction [18]. On some occasions, pterygium resection was carried out at the beginning of cataract surgery to obtain a better view inside the eye, or at the end of the surgery. At the end of cataract surgery, a trabeculotomy in ab interno approach was performed with a spatula-shaped microhook (Tanito) under the visualization of trabecular meshwork by a gonioprism in the condition of hyaluronan being filled in the anterior chamber just after the intraocular lens was implanted [19].

A major limitation in this study is the fact that the number of patients with cataract surgeries involved in the study was small, due to the extremely low incidence of endophthalmitis after cataract surgeries in general. A series of cataract surgeries performed by a single surgeon in a single institution in this study would be a merit of the study design, but a limitation when generalizing the present results to cataract surgeries performed by other surgeons in other institutions. Even with these limitations, the current results offer evidence for eye doctors to stop using prophylactic systemic antibiotics at cataract surgeries in Japan where judicial precedents have distorted the flow of changing medical practices.

## 5. Conclusions

This retrospective study reviewed the real-world data of consecutive cataract surgeries in a single institution performed by a single surgeon as showing no postoperative surgical site infection, irrespective of the use or lack of use of prophylactic systemic antibiotics. The administration of prophylactic systemic antibiotics would not be required in cataract surgeries at local anesthesia under the condition that prophylactic topical antibiotics eye drops are used and the ocular surface at the surgery is frequently irrigated with saline-diluted povidone iodine. It is recommended to share these local precautionary measures at all institutions where eye surgeries are performed. The generalizability of the present study results should be corroborated in future studies with different situations.

## Figures and Tables

**Table 1:** The number of cataract surgeries in each stage.

Stage	Period	Intravenous Antibiotics on Surgery Day	Oral Antibiotics on Surgery Day	Oral Antibiotics on the Next 2 Days	Number of Surgery	Right Eye/Left Eye	Men/ Women	Range of Age (Median)
Stage 1	April 2016–18 January 2018	Cefazolin 1 g	Cefdinir 100 mg	Cefdinir 300 mg	649	333/316	194/229	41–93 (77)
Stage 2	26 January 2018–August 2019	None	Cefdinir 200 mg	Cefdinir 300 mg	541	273/268	158/187	50–101 (77)
Stage 3	September 2019–December 2019	None	Levofloxacin 500 mg	Levofloxacin 500 mg	103	54/49	25/47	56–92 (76)
Stage 4	January 2020–October 2021	None	Levofloxacin 500 mg	None	545	270/275	144/202	45–94 (77)
Stage 5	November 2021–October 2022	None	None	None	311	160/151	84/111	47–101 (75)

**Table 2:** Conjunctival sac culture in each period.

Stage	Stage 1		Stage 2		Stage 3		Stage 4		Stage 5	
Period	April 2016–18 January 2018		26 January 2018–August 2019		September 2019–December 2019		January 2020–October 2021		November 2021–October 2022	
Right eye (R.E.) or left eye (L.E.)	R.E.	L.E.	R.E.	L.E.	R.E.	L.E.	R.E.	L.E.	R.E.	L.E.
The number of surgeries	333	316	273	268	54	49	270	275	160	151
The number of negative culture	255	238	186	182	42	37	238	242	123	130
The percent of negative culture	77%	75%	68%	68%	78%	76%	88%	88%	77%	86%
The number of positive culture	78	78	87	86	12	12	32	33	37	21
The percent of positive culture	23%	25%	32%	32%	22%	24%	12%	12%	23%	14%
Positive bacterial species										
Coagulase-negative staphylococcus (MR-CNS)	27	21	35	25	5	7	10	20	2	5
*Staphylococcus aureus* (MRSA)	2	3	2	3	1	0	5	1	0	1
MR-CNS and MRSA in total	29	24	37	28	6	7	15	21	2	6
The percent of MR-CNS and MRSA in positive culture	37%	31%	43%	33%	50%	58%	47%	64%	5%	29%
Coagulase-negative staphylococcus (MS-CNS)	47	49	45	46	11	11	23	17	8	3
*Staphylococcus epidermidis*	0	0	0	0	0	0	7	5	29	18
*Staphylococcus aureus* (MSSA)	6	13	12	8	1	1	12	14	4	5
α streptococci	8	4	13	5	0	2	4	6	1	1
β streptococci	5	5	0	0	0	0	0	0	1	0
γ streptococci	3	1	0	2	0	0	0	0	0	0
*Streptococcus pneumoniae*	1	1	2	2	0	0	1	0	2	1
*Streptococcus agalactiae* (Group B)	1	1	0	0	0	0	0	0	0	0
*Streptococcus dysgalactiae*	0	0	2	1	0	0	1	3	0	0
*Streptococcus pyogenes* (Group A)	0	1	0	0	0	0	0	0	0	0
*Corynebacterium* species	22	14	33	26	3	4	19	20	8	5
*Enterococcus faecalis*	4	7	3	3	2	1	6	6	0	2
*Haemophilus influenzae*	2	2	0	1	0	0	0	0	0	0
*Haemophilus* species	1	0	0	0	0	0	0	1	0	0
*Morganella morganii*	0	3	1	1	0	0	0	0	1	0
*Proteus vulgaris*	1	1	0	0	0	0	0	0	0	0
*Proteus* species	0	1	0	1	0	0	1	1	0	0
*Escherichia coli*	1	1	0	0	0	0	1	0	0	0
*Enterobacter* species	0	0	1	1	0	1	0	0	0	0
*Chryseobacterium indologenes*	0	0	0	1	1	0	0	0	0	0
*Serratia marcescens*	1	0	0	0	0	0	0	1	0	0
*Pseudomonas aeruginosa*	0	1	0	0	0	0	1	0	0	0
*Pseudomonas* species	2	1	0	0	0	0	0	0	0	0
*Neisseria* species	0	1	0	1	0	0	0	0	0	0
*Moraxella* species	0	2	3	0	0	0	1	1	0	1
*Klebsiella pneumoniae*	0	0	1	0	0	0	0	0	0	0
*Klebsiella* species	0	1	2	1	0	0	0	1	1	0
*Citrobacter koseri*	0	1	1	0	1	0	0	0	2	1
*Bacillus cereus*	0	0	0	0	0	0	0	1	0	0
*Providencia rettgeri*	0	0	0	0	0	0	1	0	0	0
*Acinetobacter* species	0	0	1	3	0	0	1	0	0	0
*Delftia acidovorans*	0	0	0	0	0	0	0	1	0	0
*Aeromonas hydrophila*	0	0	1	0	0	0	0	0	1	0
*Pasteurella multocida*	0	0	1	0	0	0	0	0	0	0

Bacterial species in positive culture overlap in some eyes. MR, methicillin-resistant; MS, methicillin-sensitive. *Staphylococcus epidermidis* is specifically identified in coagulase-negative staphylococcus (CNS) in recent years.

## Data Availability

The original data sheet, created and analyzed in the current study, is available from the corresponding author on reasonable request.
